# A Heterogeneous Hardware Accelerator for Image Classification in Embedded Systems

**DOI:** 10.3390/s21082637

**Published:** 2021-04-09

**Authors:** Ignacio Pérez, Miguel Figueroa

**Affiliations:** Department of Electrical Engineering, Universidad de Concepción, Concepción 4070386, Chile; ignperez@udec.cl

**Keywords:** convolutional neural network, MobileNet V2, field-programmable gate array, power consumption

## Abstract

Convolutional neural networks (CNN) have been extensively employed for image classification due to their high accuracy. However, inference is a computationally-intensive process that often requires hardware acceleration to operate in real time. For mobile devices, the power consumption of graphics processors (GPUs) is frequently prohibitive, and field-programmable gate arrays (FPGA) become a solution to perform inference at high speed. Although previous works have implemented CNN inference on FPGAs, their high utilization of on-chip memory and arithmetic resources complicate their application on resource-constrained edge devices. In this paper, we present a scalable, low power, low resource-utilization accelerator architecture for inference on the MobileNet V2 CNN. The architecture uses a heterogeneous system with an embedded processor as the main controller, external memory to store network data, and dedicated hardware implemented on reconfigurable logic with a scalable number of processing elements (PE). Implemented on a XCZU7EV FPGA running at 200 MHz and using four PEs, the accelerator infers with 87% top-5 accuracy and processes an image of 224×224 pixels in 220 ms. It consumes 7.35 W of power and uses less than 30% of the logic and arithmetic resources used by other MobileNet FPGA accelerators.

## 1. Introduction

Convolutional neural networks (CNNs) [1] have been widely used in object detection, image classification, and semantic segmentation because of their high accuracy. Compared to other image-classification techniques such as Local Binary Patterns (LBP), Scale Invariant Feature Transform (SIFT), K-Nearest Neighbor (KNN) or Support Vector Machines (SVM), CNNs show better robustness when performing classification with large databases, achieving better accuracy in their results [2,3,4]. Therefore, CNNs have occupied a fundamental role in the development of different applications, such as video surveillance [5], autonomous and assisted driving [6], assistance navigation for blind and visually impaired people [7], detection of defects in structures [8], and clinical assistance [9,10]. CNNs perform image classification during inference, a process that depends on the architecture of the network and yields different results depending on the CNN type. In particular, MobileNet [11,12] is a CNN architecture that features similar accuracy to VGG, GoogLeNet, and ResNet [13,14,15], and better accuracy compared to AlexNet and SquezeNet [16,17], while using fewer parameters than these other networks.

Even with the reduced number of parameters of MobileNet, inference is a computationally expensive process because it performs a large number of mathematical operations on the input and intermediate data, and requires fast access to a large number of parameters. In many cases, this inference is performed online on the input data. A notable case is embedded systems, which frequently operate on images or video acquired from their environment, and uses inferences to make decisions in real time [18]. Because embedded processing hardware faces severe restrictions in computational resources and power consumption, performing CNN inference in real time is a challenging task.

Graphics processing units (GPUs) are an attractive platform to implement CNN inference with high performance because they can exploit the large data parallelism available in these algorithms to perform more than one order of magnitude faster than traditional processors [19,20]. However, they reach power consumptions of up to 200 W [21], making it difficult to use them in portable and mobile devices [22]. Embedded GPUs use custom acceleration, such as the Nvidia TensorRT environment in the Jetson family, to reduce power consumption compared to traditional GPUs [23], but their power consumption is still high compared to dedicated hardware solutions on reconfigurable hardware platforms such as field-programmable gate arrays (FPGAs) [24]. Dedicated hardware accelerators for neural networks, such as Google Coral [25], offer very good performance and power efficiency, but their architecture limits their programmability and the ability to dynamically retarget the hardware for other tasks in a video-processing pipeline.

FPGAs are hardware platforms that can implement custom architectures for a wide variety of algorithms with a high level of fine-grained data parallelism. Moreover, because an FPGA can be dynamically reconfigured, its hardware can be shared between different tasks in an application using time multiplexing. Indeed, recent published work has shown implementations of MobileNet and other CNN inference algorithms on FPGA [26,27]. However, these implementations use large devices with relatively high power consumption that makes it difficult to use them in edge devices for applications that need to achieve a balance between energy efficiency and inference speed [28]. One such application is object or face recognition in mobile devices, where a trade-off between video frame rate and power consumption can be achieved, and often an inference speed of one frame per second (fps) is sufficient to classify all faces of interest in the input images [29]. Another example is the semantic segmentation of images in drones and nanosatellites, which favor architectures with high accuracy, a small number of parameters, and compact implementation on low-power edge devices [30].

In this paper, we describe the architecture of a heterogeneous hardware accelerator for CNN inference using the MobileNet V2 network. The architecture combines an embedded processor and reconfigurable hardware to achieve low power and resource utilization, with an inference speed suitable for most embedded video applications. Our design uses loop tiling to reuse data, pruning to eliminate parameters in CNN, and quantization to compress the parameters of the network. These techniques allow us to reduce the effective size of the CNN and efficiently implement it on an FPGA. As a result, our accelerator uses fewer on-chip memory and logic/arithmetic resources other MobileNet implementations in the literature and has lower power consumption compared to other embedded devices. We designed and implemented the accelerator using high-level synthesis (HLS) and describe the design space exploration to maximize inference performance. The main contributions of our work are:

- We designed a heterogeneous architecture on programmable hardware to accelerate MobileNet V2 inference with lower resource utilization and power consumption than other published work. This allows our design to be synthesized on edge devices for applications that favor low resource usage over high inference speed.
- We use loop tiling, loop unrolling, pruning, and quantization techniques to maximize the inference performance of the MobileNet V2 network and, at the same time, maintain low power consumption and resource usage on our accelerator.
- Our implementation on a Xilinx XCZU7EV FPGA running at 200 MHz consumes 29 times less power than a desktop processor, and 5 times less than an embedded GPU. It also uses 30% of the on-chip memory resources and 25% of the arithmetic resources of other published MobileNet FPGA accelerators.

The rest of this paper is organized as follows: Section 2 shows related work, detailing design techniques and CNN FPGA accelerators. Section 3 details the MobileNet V2 model, shows the base data for training and inferring, and explains the techniques used to reduce, accelerate, and implement the CNN in the architecture. Section 4 describes the architecture and the design space exploration of our custom CNN accelerator. Section 5 shows experimental results and compares them with other published works. Finally, Section 6 presents the conclusions and future work.

## 2. Related Work

### 2.1. CNN Inference on FPGAs

Inference process in CNNs is done in two stages: convolutional layers, which are used to extract patterns or features maps of the images, and classification layers, used to classify the features. In the convolutional stage, each layer applies *N* convolutions on the input map, where *N* is the number of channels or depth of the output map. Then, an activation function removes the pixels of the output map that are not relevant, and a reduction function reduces the size of the activation map. The CNN repeats this stage according to the model it uses. In the classification stage, the CNN linearly associates the feature maps to obtain *C* output data elements, where *C* is the number of categories that the CNN can recognize. The outputs of the classification layer represent the probability of classification of the input image in each category.

Performing CNN inference on an FPGA is a challenging problem, due to the limited logic, arithmetic, and memory resources available on the device, and the performance limitations imposed by the reconfigurable hardware [31]. CNN inference requires performing millions or billions of arithmetic operations in each layer [32]; moreover, a CNN typically uses millions of parameters making it impossible to store all weights and activations on the on-chip memory available on most FPGAs [33,34]. Therefore, most accelerators store data off chip, which increases inference time due to the limited memory bandwidth available on the device [35].

Recently, research has used different methods to solve these problems. Published work [32,36,37,38] proposes using loop tiling and loop unrolling to reuse weights and activations, reducing the size of data in on-chip memories, removing bubbles in the pipeline and parallelizing operations. Many approaches [32,39,40,41,42,43] use quantization strategies, which reduce the number of bits used to represent weights and activations. Other optimizations [44,45,46,47,48] apply pruning and fine-tuning techniques to reduce the number of parameters of the network. Because quantization and pruning reduce the size of the CNN, these techniques both speed up computation and increase fraction of the parameters that can be stored in on-chip memory.

CNN accelerators described in the literature have used the techniques listed above for CNN inference in FPGAs. For example, the architecture described in [32] implements VGG16 [13] using singular value decomposition (SVD), loop tiling, and loop unrolling, achieving inference at 4.45 fps. The authors in [39] use a design flow with quantization and pruning techniques to implement different CNNs on dedicated hardware and reach 2.75 fps in VGG16. The architecture described in [26] uses quantization, pruning, and loop tiling to store all feature maps and weights in on-chip memories, processing 32 channels in parallel on each processing element (PE) to speed up MobileNet [11] inference, and achieve 127 fps. The hardware accelerator described in [27] uses MobileNet V2 [12] with 16-bit quantization. This design stores all the feature maps in on-chip memories using a large FPGA. The architecture stores the weights in off-chip memories and transfers them to a buffer in the FPGA using direct memory access (DMA) in Scatter-Gather (SG) mode. Moreover, the accelerator uses four PEs, where each can process 32 channels in parallel, and achieves 266 fps. DiracDeltaNet [40] is based on ShuffleNet [49], but replaces convolutions with shift operations and uses PACT quantization to classify at 58.7 fps on an FPGA. The architecture described in [50] uses reverse-pruning and peak-pruning strategies to improve the compression factor in AlexNet [16] without sacrificing accuracy. The authors of [51] create a design flow to implement CNN inference in FPGA-based SoCs using high-level synthesis (HLS). The design flow uses Matlab to quantize and binarize the parameters and the algorithm of CNN. The workflow transforms the algorithm into an HLS C/C++ implementation to implement it on an FPGA. The authors use the design flow in SqueezeNet [17] and achieve a throughput of 14.2 fps.

Table 1 summarizes the results of the FPGA-based CNN implementations described above using the standard ImageNet dataset [52]. It includes the FPGA platform used, the CNN architecture and techniques used in the designs, the resource utilization, and the frame rate supported reported by the accelerator. CNN implementations with more parameters, such as AlexNet and VGG, infer each image in more time than smaller CNNs such as MobileNet V1 and V2. This is because the AlexNet and VGG implementations need to store weights and activations in off-chip memory, adding data-access latency. On the other hand, the reported MobileNet implementations operate at over 100 fps because they store all the weights and activations in on-chip memory using large FPGAs, which reduces latency and increases parallelism. However, these implementations are resource-intensive, particularly using a large number of on-chip memory blocks (BRAMs) and multiplier/adders (DSP slices), which makes it difficult to implement them on lower-end devices with limited resources. General-logic (LUT) resource usage is high on both types of CNN architectures.

### 2.2. Other Image Classification Algorithms on FPGAs

As mentioned in Section 1, algorithms like SIFT or SVM can also be used to classify images. Like CNNs, these algorithms have been implemented on FPGAs to speed up the classification. The architecture described in [53] combines SIFT to extract image features and SVM for classification. The authors implement the architecture on a Virtex 5 FPGA, using 38,000 LUTs, 9000 registers, and 52 DSP blocks. The hardware implementation can infer an image of the Caltech-256 database in 0.25 ms, 5.72 times faster than an equivalent software implementation. The hardware accelerator described in [54] uses SVM to detect melanoma on an FPGA. The authors use HLS to design an SVM classifier and implement it on an FPGA as a heterogeneous system using a Zynq-7 ZC702 evaluation board. The architecture uses 17,500 LUTs, 48 BRAMs, and 5 DSPs to classify an image in 11.46 μs, consuming 2 W of power. Although these implementations use fewer resources and are faster than CNNs, they achieve lower classification accuracy on large databases, thus limiting their applicability [2,4].

## 3. Methods

This section shows the MobileNet V2 CNN architecture, the dataset used in our evaluation, the modifications that we made to the network to implement it on dedicated hardware, and the architecture of the accelerator.

### 3.1. MobileNet V2

MobileNet V2 has two types of layers: (1) convolutional layers, grouped into bottleneck blocks that combine standard, depthwise, and expansion/projection convolutions with batch normalization and activation functions, and (2) classification layers, which use pooling and fully connected layers.

#### 3.1.1. Convolutional Layers

**Figure 1:**
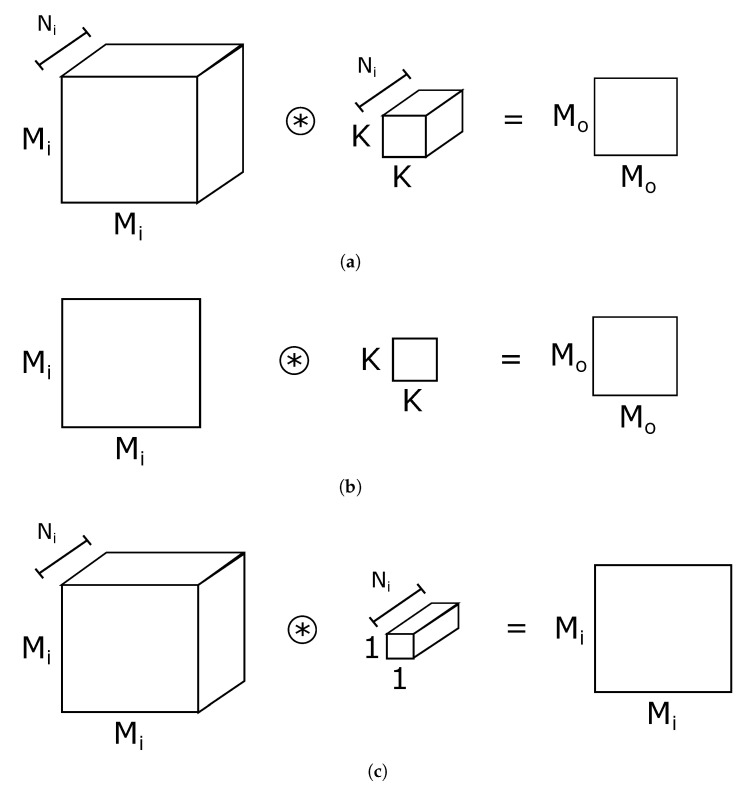
Convolution types in MobileNet V2. (**a**) standard convolution; (**b**) depthwise convolution; (**c**) expansion/projection convolution.

Figure 1 shows the standard, depthwise, and expansion/projection convolutions of MobileNet V2. $N_{i}$ and $N_{o}$ are the number of input and output channels, respectively, $M_{i}$ and $M_{o}$ are the size of the input and output feature maps, and *K* is the size of convolutional masks (K=3 in MobileNet V2). MobileNet V2 uses standard convolutions only in the first layer to combine the RGB channels of the input images. To calculate each output channel, the CNN performs the sum of the $N_{i}$ convolutions between each input channel and the corresponding convolutional mask. Depthwise convolutions use K×K=3×3 masks and a single convolution to extract features on each output channel. Expansion/projection convolutions calculate each output channel by adding the $N_{i}$ convolutions of each input channel, but replacing the 3×3 mask with 1×1 weights, transforming convolutions into multiplications. Expansion convolutions increase the number of channels, while projection convolutions reduce it.

MobileNet V2 uses batch normalization [55] to improve the speed, performance, and stability of training and inference at convolutional layers. Equation (1) shows the batch normalization:

(1) $$y_{norm}=\frac{y_{conv}-E\left[y_{conv}\right]}{\sqrt{\mathrm{Var}[y_{conv}]+\epsilon }}\times \gamma +\beta$$

where $y_{conv}$ and $y_{norm}$ are the input and output feature maps, $E[y_{conv}]$ and $\mathrm{Var}[y_{conv}]$ are the mean and variance of $y_{conv}$, γ and β are multiplicative and additive weights, and ϵ is the stability coefficient. During inference, $E[y_{conv}]$ and $\mathrm{Var}[y_{conv}]$ are constant values, which are computed during training. As an activation function, MobileNet V2 uses ReLU6(), which saturates with input values less than zero and greater than six, and helps to maintain CNN stability.

**Figure 2:**
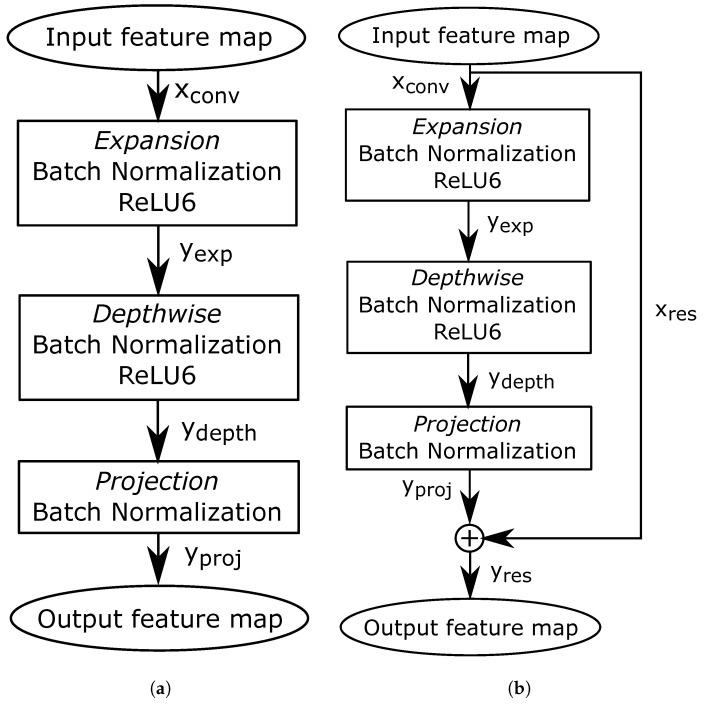
Bottleneck blocks. (**a**) bottleneck block without residual layer; (**b**) bottleneck block with residual layer.

MobileNet V2 combines convolution, normalization, and activation stages into bottleneck blocks, which are shown in Figure 2. Bottleneck blocks increase the number of channels with expansion convolutions, extract features with depthwise convolutions, and reduce the output depth with projection convolutions. Figure 2b shows a variant of a bottleneck block that uses residual layers [15]. Residuals allow the CNN to increase the number of layers, improving inference precision. These layers add the input feature map $x_{conv}$ and the output of projection convolution $y_{proj}$ to compute the output feature map $y_{res}$.

#### 3.1.2. Classification Layers

MobileNet V2 uses an average pooling function to transform three-dimensional feature maps to a one-dimensional array. The pooling function averages the pixels of each channel of the feature map to generate a vector of size $N_{o}$, which is the depth of the output feature map of the last convolution layer. Then, the CNN applies a fully-connected layer to classify the features in the array. Equation (2) shows the computation of the fully connected layer:

(2) $$y_{fc}\left[i\right]=b_{fc}\left[j\right]+\sum_{j=0}^{N_{o}-1}W_{fc}\left[i,j\right]\times y_{avg}\left[j\right]\;\mathrm{with}\;0\leq i\leq N_{class}$$

The CNN linearly associates each component of the array $y_{avg}$ with each other by adding the product between each component and weight $W_{fc}$ to the bias $b_{fc}$. The output array $y_{fc}$ represents the classification probabilities of the $N_{class}$ categories in the input image.

#### 3.1.3. MobileNet V2 Model

Table 2 shows the MobileNet V2 model. It uses a standard convolution in the first layer to combine the RGB channels, 18 bottleneck blocks to extract features, and an average pooling and a fully connected layer to classify the features. The neural network has 3.4 million parameters.

### 3.2. Imagenet Dataset

To test their performance on object recognition, CNNs typically use the ImageNet dataset [52]. ImageNet has about 1.3 million high-definition RGB images divided into 1000 categories. The dataset has a training group with 1.3 million images, a validation group with 50,000 images, and a test group with 100,000 unlabeled images, which are used to compute the accuracy of the network.

### 3.3. Complexity-Reduction Techniques

Following the experience published in the literature [36,43,46,56], we applied different techniques to MobileNet V2 to reduce its complexity before designing the architecture of the hardware accelerator. We merged batch normalization into convolutions, divided the activations and weights using loop tiling, and used pruning and quantization techniques to reduce the size of the network.

#### 3.3.1. Batch Normalization

Batch normalization is complex to implement on FPGA hardware because it requires computing a division and a square root. These operations are resource-intensive and add significant latency. To simplify the FPGA implementation, we merged batch normalization with convolutions [56], by modifying the convolutional masks and adding a bias. We factored Equation (1) into the computation of $y_{conv}$ to obtain Equation (3):

(3) $$y_{norm}=\frac{\gamma }{\sqrt{\mathrm{Var}[y_{conv}]+\epsilon }}\times y_{conv}+\left(\beta -\frac{E[y_{conv}]}{\sqrt{\mathrm{Var}[y_{conv}]+\epsilon }}\times \gamma \right)=W_{norm}\times y_{conv}+b_{norm}$$

where $W_{norm}=\frac{\gamma }{\sqrt{\mathrm{Var}[y_{conv}]+\epsilon }}$ are the weights and $b_{norm}=\left(\beta -\frac{E[y_{conv}]}{\sqrt{\mathrm{Var}[y_{conv}]+\epsilon }}\times \gamma \right)$ is the bias of the normalization. Then, we combined Equation (3) with the convolution $y_{conv}=x_{conv}\times W_{conv}$ to obtain Equation (4):

(4) $$\begin{array}{ccc} y_{norm} & = & \left(W_{norm}\times W_{conv}\right)\times x_{conv}+b_{norm}\\ y_{norm} & = & W_{cn}\times x_{conv}+b_{norm} \end{array}$$

where $x_{conv}$ and $y_{conv}$ are the input and output maps, respectively, and $W_{conv}$ are the convolutional weights. Equation (4) shows the batch normalization process folding into convolutions, with $W_{cn}$ and $b_{norm}$ being the new convolutional weights and bias, respectively.

#### 3.3.2. Loop Tiling

**Figure 3:**
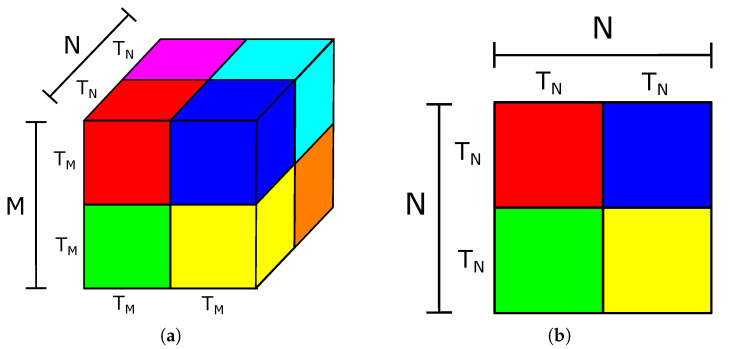
(**a**) Loop tiling in feature maps; (**b**) loop tiling in weights.

We used loop tiling to reuse the data stored on the limited on-chip memory in an FPGA. Figure 3 shows the division used on the maps and parameters to apply the technique. We separated the activations and weights into blocks of size $T_{M}\times T_{M}\times T_{N}$ and $T_{N}\times T_{N}$, where $T_{M}$ and $T_{N}$ are tiling factors used on the map size and layer depth, respectively. Each iteration of the inference computes one block at a time, reducing the number of on-chip memory blocks used in this part of the architecture.

#### 3.3.3. Pruning

**Figure 4:**
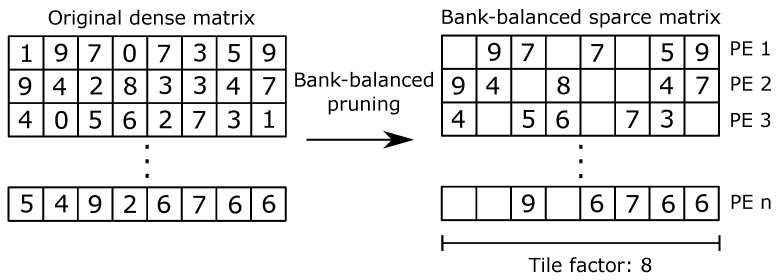
Bank-balanced pruning in MobileNet V2.

Although pruning allows us to reduce the number of weights and arithmetic operations, it must be applied carefully so that each PE processes a similar amount of data. Otherwise, load unbalance will negatively affect the performance of the accelerator. We applied the bank-balanced pruning technique proposed in [46], which groups data in blocks and uses pruning to remove the same number of parameters in each block. Figure 4 shows the bank-balanced pruning that we used on MobileNet V2. We divided the weights into blocks of size $T_{N}$, where $T_{N}$ is the tiling factor. Then, we removed the data with the lowest absolute value and retrained the CNN to improve accuracy. We repeated the process until we reached an acceptable pruning factor and inference accuracy.

Because the new post-pruning weight matrix is sparse, we used the index system presented in [44], where each non-zero weight is associated with an index that stores the distance with the next non-zero weight. Using this technique, we store only non-zero parameters in on-chip memory.

In our application, we did not apply pruning to depthwise convolution because their weights only represent 3% of the total number of MobileNet V2 parameters. Moreover, because depthwise convolution extracts features in the CNN, removing weights from that layer negatively affects accuracy. Specifically, applying a reduction rate of 0.1 in depthwise convolution layers only eliminates 0.19% of the total weights of the network, but top-1 accuracy reduces by 16.67%. Conversely, when we apply the same reduction rate to expansion/projection the convolution layers, we can eliminate 6.25% of the total parameters, and top-1 accuracy is reduced only by 0.84%.

#### 3.3.4. Quantization

We used dynamic quantization [43] to reduce the number of bits in the activations and weights. Equation (5) shows the quantization strategy that we applied to MobileNet V2. We divided the floating-point data $y_{float}$ by a scaling factor Δ and rounded the result to obtain the quantized fixed-point value $y_{quant}$. The scaling factor Δ is computed as the difference between the maximum and minimum floating-point data divided by number of values that can be represented by $y_{quant}$:

(5) $$y_{quant}=\mathrm{round}\left(\frac{y_{float}}{\Delta }\right)\;\mathrm{with}\;\Delta =\frac{\max\left(y_{float}\right)-\min\left(y_{float}\right)}{2^{bits}}$$

## 4. Hardware Architecture

### 4.1. MobileNet V2 Accelerator Architecture

#### 4.1.1. General Architecture

**Figure 5:**
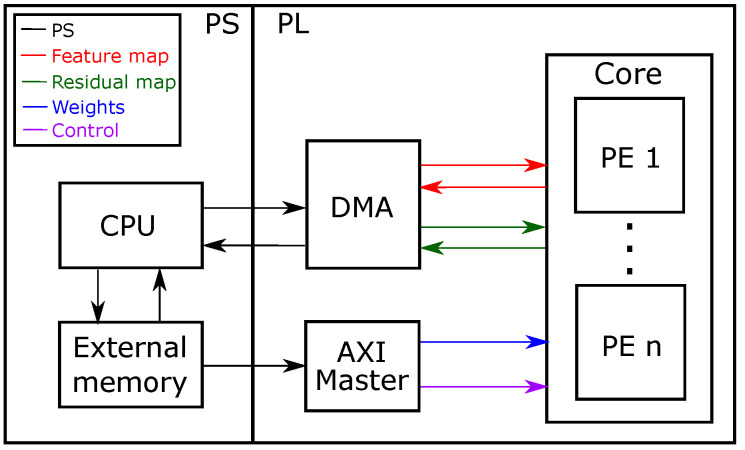
Architecture of the MobileNet V2 accelerator.

Figure 5 shows the architecture of our MobileNet V2 accelerator. The heterogeneous architecture is divided into a processing system (PS) and programmable logic (PL). The PS integrates a programmable processor (CPU) that acts as a controller and off-chip memory that stores the activations and weights of the CNN. The PL uses reconfigurable logic to implement the PEs that compute each layer of the network. In our current implementation, the PL has four PEs that operate in parallel. Inference in the architecture operates as follows: (1) the CPU writes the off-chip memory address of weights and control data onto the PL using an AXI Master protocol, (2) the CPU sends feature and residual maps from external memory to each PE as a data stream, using a direct-memory access (DMA) controller, (3) each PE stores the blocks of weights and control data in on-chip buffers, and (4) the PEs compute each layer, reading and writing the input and output activations from/to off-chip memory as a data streams using DMA. The accelerator iterates over the data until it completes the inference.

#### 4.1.2. Processing Elements

Each PE contains functional units (FUs) that are custom-designed to compute each type of MobileNet V2 layer. A multiplexer selects the correct FU at each point in the computation.

**Figure 6:**
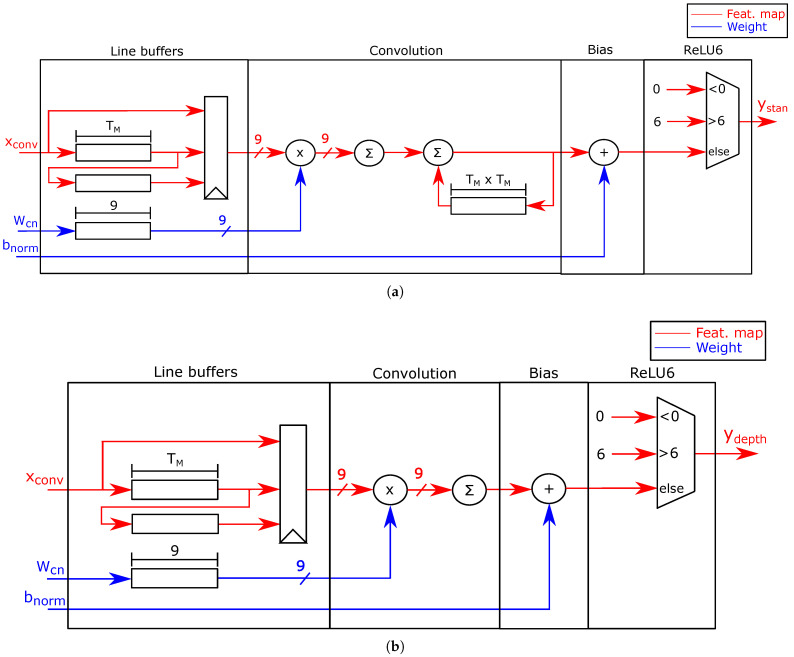
(**a**) Standard convolution FU; (**b**) depthwise convolution FU.

Standard and depthwise FUs: Figure 6 shows the FUs that computes the standard and depthwise convolutions. The input maps are stored in line buffers. The standard and depthwise FUs implement Equation (4) to compute the convolutions. The FUs also use a multiplexer to implement ReLU6 function. The PEs write out each output map as a data stream onto external memory.

Standard and depthwise convolutions use values in a 3×3 neighborhood to compute their results. Because the PEs receive the pixels as a data stream that traverses consecutive rows of the tiling map, the FUs use two line buffers and a 9-register array to compute the 3×3 window of the input map $x_{conv}$, and nine registers to store the 3×3 window of weights $W_{cn}$. The accelerator implements each line buffer as a First-In-First-Out (FIFO) queue using on-chip memory. Each line buffer has a size of $T_{M}$ words to store a row of the tiling map.

The second stage of the standard and depthwise FUs computes the multiplications of convolutions using nine parallel multipliers and a pipelined adder tree. The standard convolution uses an additional stage to add the convolution of the current input channel with the partial sum of the previous input channels to compute the output channel. The architecture stores the partial sum in a buffer of size $T_{M}\times T_{M}$.

The final two stages add the batch normalization bias $b_{norm}$ of Equation (4) and compute the activation function. A multiplexer implements the ReLU6 function by saturating the output to zero or six. The PEs send their outputs to external memory as a data stream.

**Figure 7:**
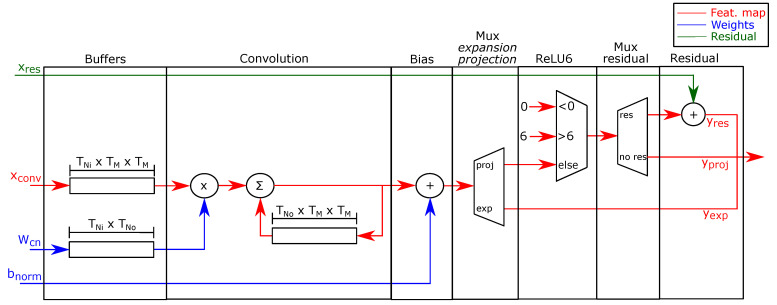
Expansion/projection FU.

Expansion/projection FU: Figure 7 shows the expansion/projection FU. Because expansion/projection convolutions reuse the input maps and use pruning to compute Equation (4), and because the CPU sends the activations only once, the FU must store the inputs. For this reason, the input stage of the FU uses buffers of size $T_{Ni}\times T_{M}\times T_{M}$ and $T_{Ni}\times T_{No}$ to store an input map block $x_{conv}$ and a weight block $W_{cn}$, respectively.

The second stage of the FU computes the convolution using the weights $W_{cn}$ and the input maps $x_{conv}$ read from the buffer. A single scalar multiplier compute the 1×1 convolutions used by these layers. Then, the FU adds the convolution of the current input channel to the partial sum of the previous channel to compute the output channel. The accelerator uses a buffer of size $T_{No}\times T_{M}\times T_{M}$ to store the partial sum.

The third stage adds the batch normalization bias $b_{norm}$ of Equation (4). If the layer is a projection convolution, the PE computes the ReLU6 activation function and adds the residual map $x_{res}$ if the layer uses it. Finally, the expansion/projection accelerator sends the output map $y_{layer}$ to external memory as a data stream.

**Figure 8:**
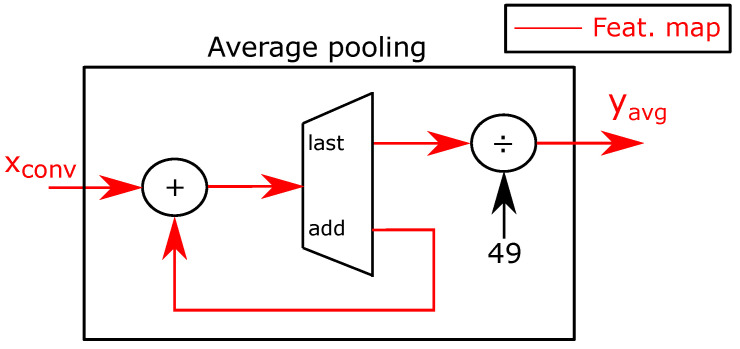
Average pooling FU.

Average pooling FU: Figure 8 shows the average pooling FU. The PE receives the input map block as a data stream. Because the CPU sends each input channel consecutively, the accelerator does not need to store the activations in buffers. The PE adds all input pixels of the current channel and stores the intermediate results in a register. Then, the accelerator divides the sum by the number of pixels of the channel to compute the average value. Because the last convolutional map is of size 7×7, the accelerator divides the sum by the constant value 49. To simplify the design, we used a lookup table (LUT) to implement the division. Finally, the FU streams out the output $y_{avg}$ to external memory.

**Figure 9:**
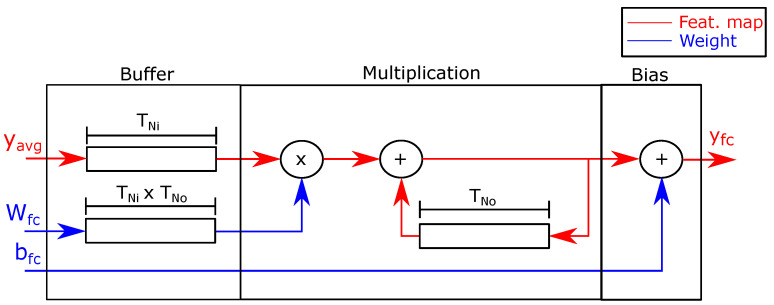
Fully-connected layer FU.

Fully-connected layer FU: Figure 9 shows the architecture of the fully-connected layer FU. An input stage stores the input array and weights in buffers. The PE computes Equation (2) and streams the output array to the CPU using DMA.

Like the average pooling FU, the fully-connected layer reuses the input array block read from the CPU $N_{o}$ times to compute Equation (2). Therefore, each PE stores a full tile of the input in a buffer of size $T_{Ni}$. Similarly, the accelerator stores the weight blocks $W_{fc}$ in a buffer of size $T_{Ni}\times T_{No}$ to apply the pruning indices.

The second stage computes Equation (2) using the input array $y_{avg}$, the weights $W_{fc}$, and the bias $b_{fc}$. The PE multiplies the input $y_{avg}$ by the weight $W_{fc}$ and adds the output to the partial sum of the previous results of the current component of the output array $y_{fc}$. The architecture uses a buffer of size $T_{No}$ to store the partial results. Finally, when the FU has added the $N_{o}$ products, it adds the bias $b_{fc}$ and streams the output to the CPU, which writes it out as the inference result.

#### 4.1.3. Parallel Map Processing

**Figure 10:**
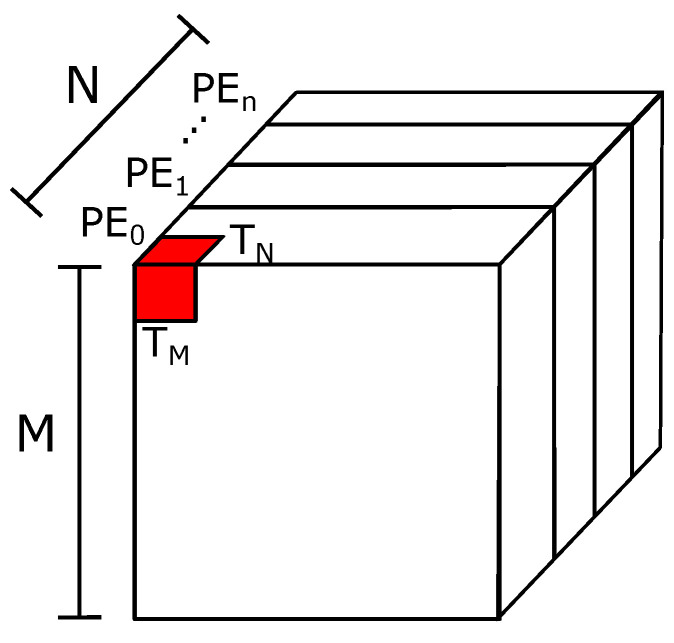
Partition of feature maps for parallel PE processing.

Our architecture uses n=4 PEs in parallel to speed up the inference. Figure 10 shows the spatial division of feature maps, where each partition is computed in a separate PE. We partition the maps across the channels to keep the bank-balanced pruning intact. Each PE processes N/n channels, subdivided into blocks of size $T_{N}$ for each iteration of the layer.

### 4.2. Design Space Exploration

In this section, we show how we applied loop tiling, pipelining, loop unrolling, and array partitioning to improve the performance of the accelerator.

#### 4.2.1. Loop Tiling Factor

As discussed in Section 3.3.2, we used loop tiling to the depth and size of the data. We considered different loop tiling factors $T_{N}$ and $T_{M}$ to reduce inference time. Table 3 shows the inference time for different loop tiling factors applied to activations and weights. The times were measured on the implementation of the accelerator described in Section 5. As the table shows, inference time decreases with the size of the loop tiling factor because the accelerator reuses more data, decreasing access to off-chip memory. However, larger tiling factors require more on-chip memory. We used loop tiling factors of 32 and 28 for the depth and size, respectively, which provide and adequate trade-off between performance and memory usage adequate for limited-resource devices.

#### 4.2.2. Pipelining

The accelerator combines the spatial parallelism of multiple PEs with deep pipelining within the architecture of each PE. A pipelined architecture executes multiple stages of the computation in parallel on different data, using synchronized registers to decouple the stages. Although latency is not decreased and can even increase in a pipelined architecture, throughput and clock rate increase, boosting performance. We use pipelining in the design of every FU in the PEs.

#### 4.2.3. Loop Unrolling Factor

The loop unrolling technique eliminates bubbles caused by control dependencies in the pipeline and exposes additional parallelism in the algorithm. Although the data stream provided by the DMA controller restricts parallelism because the PEs can only access one data element at a time, in the expansion/projection accelerator, we can parallelize its operation when the PE reads the data from the activation buffer. For this reason, we used loop unrolling on the inner loop that traverses the feature map block. Table 4 shows the number of clock cycles needed by the accelerator to execute the inner loop of the expansion/projection convolution, for different loop unrolling factors. As the table shows, processing time decreases with the unrolling factor because it increases the data parallelism available to the PE. We used a loop unrolling factor of 28 in the inner loop of expansion/projection convolution FU. Larger unrolling factors have no effect because performance is limited by the loop tiling factor.

#### 4.2.4. Array Partitioning

HLS allows using an array partitioning pragma to divide an array into blocks and synthesize it in independent on-chip memories. Using this feature, the architecture can access more data at the same time, increasing parallelism. We used a partitioning factor of 28 in the map-size component of the activation buffer of the expansion/projection accelerator to access 28 pixels in parallel, which are then processed by the parallel units created through loop unrolling.

## 5. Results

### 5.1. Classification Performance

As discussed in Section 3.3, we used pruning and quantization to obtain a reduced version of MobileNet V2, and retrained the network after applying these techniques using the original parameters as initial values. Table 5 shows the MobileNet V2 hyperparameter values used for retraining and inference. We configured the network for input images of 224×224 pixels with a width multiplier of 1, which preserves the number of channels in the default configuration. The CNN has 21 layers, composed of one standard convolution layer and 18 bottleneck blocks to extract features, plus one average pooling and one fully connected layer to classify the features. For bank-balanced pruning, we used a reduction rate of 0.3 for the expansion/projection convolutions and 0.7 for the fully-connected layer, and retrained the CNN with a learning rate of 0.001, a momentum of 0.9 and 30 epochs. In dynamic quantization, we used 12 and 10 bits for maps and parameters in the convolutional layers, respectively, and 12 and 6 bits for activations and weights in the fully-connected layer. With these modifications, our reduced version of MobileNet V2 achieves top-1 and top-5 accuracy of 65.62% and 87.03% on ImageNet. Compared to the unmodified network, top-1 and top-5 accuracy is reduced by 6.26% and 3.26%, respectively. Table 6 shows examples of inference results in our reduced CNN. Columns 2 and 3 of the table show images that the network correct classifies in the top and within the top 5 probabilities, respectively, while column 4 shows an incorrectly classified image.

### 5.2. Performance and Resource Utilization

We designed the accelerator architecture using the Xilinx HLS environment. We used Xilinx Vivado HLS 2019.2 to compile the HLS code to a register transfer level (RTL) description, and Xilinx Vitis IDE 2019.2 to manage the CPU and processor-logic communication. We synthesized the design and analyzed its performance using the Xilinx Vivado 2019.2 design suite. We implemented the accelerator on the reconfigurable logic of a Xilinx Zynq UltraScale+ XCZU7EV FPGA, which also has an ARM Cortex-A53 processor. Our architecture uses four parallel PEs implemented on the FPGA, which communicates with the ARM processor using a direct memory access (DMA) block.

Table 7 shows the resource utilization of each PE in our implementation. The table separates the resources used for standard convolutions, depthwise convolutions, expansion/projection convolution, average pooling, and fully-connected FUs, the read-control information section, and the communication protocols of input and output signals on the PE. As the table shows, each PE uses 125 BRAMs and 85 DSP slices. The expansion/projection architecture uses most of the BRAMs because these layers store a full input map block. In addition, standard convolution and fully-connected FUs use BRAMs to store their partial sums. The control information section uses the rest of the BRAMs as a buffer to store the PE control signals. Because each map block is of size 28×28, standard and depthwise architectures use LUTs and registers to implement the line buffers. The expansion/projection and fully-connected FUs use 72-bit wide URAM memory blocks to store the prune weights.

Table 8 shows the resource utilization of the complete accelerator on the FPGA, using four PEs in parallel. As the table shows, this implementation of our architecture uses 532 BRAMs, 24 URAMs, and 340 DSP slices. The PEs use the URAM and DSP slices to compute the inference, while the DMA block uses 32 BRAMs as buffers to store the input and output data.

Our implementation runs at a maximum clock frequency of 200 MHz. The critical path that limits the clock frequency runs from the output of a DSP slice that computes the multiplication between the input maps and the weights, to the input of a register that stores the result in the standard convolution FU.

At the maximum clock frequency, Xilinx Vivado estimates the power consumption of the accelerator as 7.35 W, with 0.73 W and 6.62 W of static and dynamic power respectively. The AMR processor consumes 2.79 W (0.12 W of static power and 2.67 W of dynamic power), while the FPGA logic section consumes 4.56 W (0.62 W of static power and 3.94 W of dynamic power). Each PE consumes 0.56 W, while the DMA block and the clock distribution network consume the remaining 2.32 W.

Our implementation processes an image of 224×224 pixels at 4.54 fps (220.5 ms), performing 2.76 GOPS. The processor uses 57.4 ms to manage and send the data to the accelerator, which computes the inference in 163.1 ms. Table 9 shows the execution time of each layer of the MobileNet V2 network. As the table shows, the PS processing time is larger in the first layers of the CNN because the processor must manage and send more blocks to the accelerator when the size of the feature maps is larger than 28. The accelerator processing time increases with the size (in the first layers) or depth (in the last layers) of the activation because the number of blocks that each PE processes per iteration increases.

### 5.3. Scalability

When we synthesize the architecture onto a larger device, we can increase the number of PEs and the amount of activation and weight data that can be stored on chip. This allows the architecture to scale its performance at the cost of increased resource utilization. Table 10 shows resource utilization, inference time, and maximum fps achieved by multiple versions of the accelerator configured with 4, 8, and 12 PEs. We obtained these results by synthesizing the accelerator onto a XCZU19EG FPGA, which features with 2.26 times more LUTs and registers, 3.15 times more BRAMs, 1.33 times more URAMs and 1.14 times more DSP than our XCZU7EV FPGA. As the table shows, LUTs and BRAMs limit the number of PEs that can be implemented on the chip because the architecture requires more finite-state machines to control the inference, and stores more data in on-chip memories, respectively. With 12 PEs, the accelerator computes inference on an image in 40.65 ms, reaching a throughput of 24.6 fps.

### 5.4. Discussion

Table 11 summarizes the key parameters of the FPGA accelerators of Table 1 and compares them to the accelerator presented in this paper. As the table shows, our design is closest to [26,27], which implement versions 1 and 2 of MobileNet, respectively. Compared to our implementation, these designs achieve significantly higher throughput, mainly because they use large FPGA devices that allow them to store all the map data in on-chip memory. This has several benefits: it allows them to increase parallelism through more PEs, it eliminates the need to access comparatively slow off-chip memory, and it eliminates the computation and communication latency introduced by the processor. The cost is increased resource utilization: indeed, our design uses 23–30% of the arithmetic and memory resources used by these solutions, allowing us to map the accelerator onto a wider range of devices. Even when using 12 PEs in parallel, our accelerator exhibits lower resource usage because we process only one channel at a time in each PE to save hardware resources and power, while the other solutions process 32 simultaneous channels per PE.

Compared to the VGG [32,39] accelerators, our implementation achieves similar inference time and top-5 accuracy, but lower resource utilization. The accelerator in [40] uses the CNN DiracDeltaNet architecture, which has been custom-designed for FPGAs, replacing multiplications with shift operations and using aggressive quantization. They report better resource utilization than our implementation, with slightly better accuracy. The AlexNet accelerator [50] computes inference 2.14 times faster than our accelerator but uses a clock frequency 1.5 times faster. In comparison, our accelerator achieves better accuracy, uses less resources, and consumes 2.4 times less power. The SqueezeNet [51] CNN has fewer parameters and layers than MobileNet V2, and their accelerator is faster than our design, but with lower top-1 and top-5 accuracy.

We also compared the performance of our accelerator to the Nvidia Jetson AGX Xavier benchmarks [57] and the Google Coral Dev Board [25], which are commercially available platforms. In the case of the Nvidia Jetson platform, the processor and GPU cores on this device run at a clock frequency 2.27 GHz and 1.38 GHz, respectively. Using the ResNet-50 [15] and VGG19 CNN architectures, the GPU performs an inference between 6.8–11.3 times faster than our accelerator. However, its power consumption is 4.2–5.2 times higher, limiting its use in power-constrained edge devices. The Coral Dev Board uses a Tensor Processing Unit (TPU), a custom-built accelerator for the TensorFlow framework. Implementing MobileNet V2, the Coral hardware performs inference at 385 fps and consumes 5.4 W. Indeed, Coral achieves better performance and power than our accelerator, but its special-purpose architecture limits its application to neural-network computation. In comparison, our accelerator was designed for programmable hardware platforms, which can easily assign their logic resources to other tasks, such as image processing and machine vision algorithms, through dynamic reconfiguration.

We also compared the performance of the accelerator to desktop-class hardware. We computed MobileNet V2 inference in software using the PyTorch framework with CUDA 10.2 and tested the software on an Nvidia RTX 2080 GPU and an Intel i9-9900K CPU. The desktop GPU is one order of magnitude faster than our accelerator (21.7 ms inference), but with 30 times the power consumption (215 W). The CPU achieves approximately the same inference time (222 ms) with more than one order of magnitude higher power (95 W).

Although other accelerator architectures achieve faster inference than our design, our accelerator achieves a good balance between accuracy, performance, and power consumption. This makes our architecture attractive for embedded and portable devices that process video with limited hardware resources and power budget. For example, our accelerator could be embedded on a smart camera architecture, allowing it to classify objects at a frame rate of 4 to 25 fps, depending on the device used in the implementation. For applications such as face recognition in video analytics, this performance can be sufficient in most scenarios [29].

## 6. Conclusions

In this paper, we proposed the architecture of a hardware accelerator for MobileNet V2 inference. Our architecture was designed for reconfigurable logic devices, and features good scalability, as well as lower resource utilization and power consumption compared to other published and commercial accelerators that run on reconfigurable or programmable hardware. This allows our architecture to be implemented on edge devices for real-time image classification that are resource- and power-constrained. The accelerator uses loop tiling, bank-balanced pruning, dynamic quantization, and off-chip storage to increase performance and reduce hardware resource utilization and power consumption. Our implementation uses an embedded processor and external memory to control the execution of the algorithm and to store parameters, respectively. The accelerator exploits the parallelism available in the various MobileNet V2 layers using pipelining and multiple processing elements implemented on reconfigurable hardware. By configuring the number of processing elements, our architecture can trade off inference speed for power and resource utilization. This allows us to target the implementation to a wide range of devices, or to share hardware resources with other algorithms on the same device.

We implemented a prototype of the architecture on a Xilinx Zynq Ultrascale+ XCZU7EV FPGA with 2 GB of DDR4 external memory. Our accelerator performs inference on 224×224-pixel images at 4.53 fps, consumes 7.35 W, and achieves a top-1 and top-5 accuracy of 65.62% and 87.03%, respectively. Our implementation uses 20% and 85% of the DSP slices and on-chip memory blocks available on the FPGA. Implemented on a larger XCZU19EG FPGA, our design reaches 24.6 fps with 12 PEs.

We are currently working on improving the communication between the processor and the accelerator using a faster DMA-SG interface, which stores transfer instructions in on-chip memory instead of the CPU cache. We are also working on quantizing MobileNet V2 with PACT functions to further reduce the number of bits used to represent weights and activations, allowing us to store more data in the same amount of on-chip memory.

## Figures and Tables

**Table 1:** FPGA-based CNN inference described in the literature (N/A: Not available).

	Qiu et al. [32]	Guo et al. [39]	Su et al. [26]	Bai et al. [27]	Yang et al. [40]	Zhang et al. [50]	Panagiotis et al. [51]
Year	2016	2018	2018	2018	2019	2019	2020
FPGA	XC7Z045	XC7Z045	XCZU9EG	Arria 10 SX	XCZU3EG	XCZU7EV	XC7Z020
Freq. (MHZ)	150	214	150	133	250	300	100
CNN	VGG-16	VGG-16	MobileNet	MobileNet V2	DiracDeltaNet	AlexNet	SqueezeNet
Reduction	SVD	N/A	Pruning	N/A	N/A	Pruning	N/A
Quantization	16 bits	8 bits	8–4 bits	16 bits	1–4 bits	8 bits	8 bits
LUT	182,616	29,867	139,000	163,506	24,130	101,953	34,489
BRAM	486	85.5	1729	1844	170	198.5	97.5
FF	127,653	35,489	55,000	N/A	29,867	127,577	25,036
DSP	780	190	1452	1278	37	696	172
Power (W)	9.63	3.5	N/A	N/A	5.5	17.67	N/A
Perf. (GOPS)	136.97	84.3	91.2	170.6	47.09	14.11	N/A
fps	4.45	2.75	127	266	58.7	9.73	14.2
Top-1	N/A	67.72%	64.6%	71.8%	70.1%	55.99%	56.94%
Top-5	86.66%	88.06%	84.5%	91.0%	88.2%	N/A	79.94%

**Table 2:** MobileNet V2 model.

Input (${\mathrm{MxMxN}}_{i}$)	Output (${\mathrm{MxMxN}}_{o}$)	Layer	Repeat Time	Expansion Factor	Stride	Residual	Thousands of Parameters
224 × 224 × 3	112 × 112 × 32	Standard conv	1	-	2	No	0.8
112 × 112 × 32	112 × 112 × 16	Bottleneck	1	1	1	No	0.7
112 × 112 × 16	56 × 56 × 24	Bottleneck	2	6	2	Yes	12.9
56 × 56 × 24	28 × 28 × 32	Bottleneck	3	6	2	Yes	37.3
28 × 28 × 32	14 × 14 × 64	Bottleneck	4	6	2	Yes	177.9
14 × 14 × 64	14 × 14 × 96	Bottleneck	3	6	1	Yes	296.0
14 × 14 × 96	7 × 7 × 160	Bottleneck	3	6	2	Yes	784.2
7 × 7 × 160	7 × 7 × 320	Bottleneck	1	6	1	No	469.4
7 × 7 × 320	7 × 7 × 1280	Expansion conv	1	-	1	No	409.6
7 × 7 × 1280	1 × 1 × 1280	Avg pooling	1	-	-	-	0.0
1 × 1 × 1280	1 × 1 × 1000	FC	1	-	-	-	1280.0

**Table 3:** Inference time with different loop tiling factors.

Loop Tiling Factor		Inference Time (ms)
Activations	Weights	
14	16	252.8
14	32	239.7
14	64	241.6
28	16	230.2
28	32	220.5

**Table 4:** Processing time on expansion/projection FU with different loop unrolling factors.

Loop Unrolling Factor	Processing Cycles	
	Min	Max
Without unrolling	53	788
Unrolling ×7	30	114
Unrolling ×14	15	57
Unrolling ×28	8	29

**Table 5:** MobileNet V2 hyperparameters used for retraining and inference (N/A: not applicable).

Hyper Parameter	Retraining	Inference
Batch size	32	1
Input image size	224×224	224×224
Width multiplier	1	1
Number of layers	21	21
Learning rate	0.001	N/A
Momentum	0.9	N/A
Number of epochs	30	N/A

**Table 6:** Inference results in our reduced MobileNet V2 network (Images taken from the ImageNet 2012 validation dataset [52]).

Image id	00041633	00031834	00023151
Correct label	mink	walker hound	combination lock
Results	mink: 71.63%	beagle: 28.18%	syringe: 7.65%
	weasel: 11.72%	walker hound: 14.47%	reel: 6.17%
	polecat: 6.79%	basset: 6.34%	fountain pen: 6.00%
	hamster: 2.12%	basenji: 5.90%	corkscrew: 3.91%
	wombat: 1.91%	pembroke: 4.81%	wine bottle: 3.34%

**Table 7:** Resource utilization of each PE.

Module	Slice	Slice	BRAM	URAM	DSP
	LUT	Registers			
Standard FU	2378	1737	6	0	21
Depthwise FU	1904	1747	0	0	18
Expansion/projection FU	3668	1335	112	3	40
Average pooling FU	363	209	0	0	1
Fully-connected FU	775	578	1	3	4
Read control information	3215	2211	6	0	1
Communication protocols	3069	5514	0	0	0
Total	15,372	13,331	125	6	85
Percent	7.36%	3.06%	19.71%	6.25%	4.92%

**Table 8:** Resource utilization of the accelerator.

Module	Slice	Slice	BRAM	URAM	DSP
	LUT	Registers			
DMA	57,072	75,322	32	0	0
PEs	61,161	53,292	500	24	340
Total	118,233	128,614	532	24	340
Percent	51.32%	27.91%	85.26%	25.00%	19.68%

**Table 9:** Execution time of each layer of MobileNet V2 on the accelerator.

Input ($M_{x}M_{x}N_{i}$)	Output ($M_{x}M_{x}N_{o}$)	Layer	Processor [ms]	Accelerator [ms]	Total [ms]
224 × 224 × 3	112 × 112 × 32	Standard conv	1.5	18.1	19.6
112 × 112 × 32	112 × 112 × 16	Bottleneck	5.7	7.2	12.9
112 × 112 × 16	56 × 56 × 24	Bottleneck	29.0	29.9	58.9
56 × 56 × 24	28 × 28 × 32	Bottleneck	9.2	12.7	21.9
28 × 28 × 32	14 × 14 × 64	Bottleneck	4.0	12.9	16.9
14 × 14 × 64	14 × 14 × 96	Bottleneck	3.4	18.7	22.1
14 × 14 × 96	7 × 7 × 160	Bottleneck	3.3	30.1	33.4
7 × 7 × 160	7 × 7 × 320	Bottleneck	0.7	14.6	15.3
7 × 7 × 320	7 × 7 × 1280	E × pansion conv	0.2	12.9	13.1
7 × 7 × 1280	1 × 1 × 1280	Avg pooling	0.2	0.3	0.5
1 × 1 × 1280	1 × 1 × 1000	Fc	0.2	5.7	5.9
MobileNet V2			57.4	163.1	220.5

**Table 10:** Resource utilization and inference time on an XCZU19EG FPGA for different numbers of PEs.

Number	Slice	Slice	BRAM	URAM	DSP	Time	fps
of PEs	LUT	Registers				[ms]	
4	121,689	127,020	524	24	340	126.91	7.87
8	240,660	250,278	1047	48	680	63.45	15.76
12	368,936	391,517	1572	72	1020	40.65	24.60

**Table 11:** Comparison to other CNN accelerators (N/A: Not available).

Implementation	CNN	Top-1 (%)	Top-5 (%)	Power (W)	fps	LUT	BRAM	FF	DSP
Qiu et al. [32]	VGG-16	N/A	86.7	9.63	4.45	182,616	486	127,653	780
Guo et al. [39]	VGG-16	67.7	88.1	3.5	2.75	29,867	85.5	35,489	190
Su et al. [26]	MobileNet	64.6	84.5	N/A	127	139,000	1729	550,00	1452
Bai et al. [27]	MobileNet V2	71.8	91.0	N/A	266	163,506	1844	N/A	1278
Yang et al. [40]	DiracDeltaNet	70.1	88.2	5.5	58.7	24,130	170	29,867	37
Zhang et al. [50]	AlexNet	56.0	N/A	17.67	9.73	101,953	198.5	127,577	696
Panagiotis et al. [51]	SqueezeNet	56.9	79.9	N/A	14.2	34,489	97.5	25,036	172
This work	MobileNet V2	65.6	87.0	7.35	4.54	118,233	532	128,614	340

## Data Availability

The experiments presented in this paper were performed using images from the ImageNet database available at http://www.image-net.org/index accessed on 28 December 2020.

## Abbreviations

The following abbreviations are used in this manuscript:

BRAM	Block Random Access Memory
CNN	Convolutional Neural Network
CPU	Central Processing Unit
DMA	Direct Memory Access
DSP	Digital Signal Processor
FIFO	First In First Out
FPGA	Field-Programmable Gate Array
fps	frames per second
FU	Functional Unit
GOPS	Giga Operations per Second
GPU	Graphics Processing Unit
HLS	High-Level Synthesis
KNN	K-Nearest Neighbor
LBP	Local Binary Patterns
LUT	Lookup Table
PE	Processing Element
PL	Programmable Logic
PS	Processing System
SG	Scatter Gather
SIFT	Scale Invariant Feature Transform
SVD	Singular Value Decomposition
SVM	Support Vector Machines
RTL	Register Transfer Level
TPU	Tensor Processing Unit
URAM	Ultra Random Access Memory
